# Dendritic cell-mediated HIV-1 transmission to T cells of LAD-1 patients is impaired due to the defect in LFA-1

**DOI:** 10.1186/1742-4690-3-75

**Published:** 2006-11-01

**Authors:** Fedde Groot, Taco W Kuijpers, Ben Berkhout, Esther C de Jong

**Affiliations:** 1 Department of Human Retrovirology, Academic Medical Centre, University of Amsterdam, Amsterdam, The Netherlands; 2 Department of Cell Biology and Histology, Academic Medical Centre, University of Amsterdam, Amsterdam, The Netherlands; 3 Division of Pediatric Hematology, Immunology and Infectious Diseases, Emma Children's Hospital, Academic Medical Centre, University of Amsterdam, Amsterdam, The Netherlands; 4 Present address: The Sir William Dunn School of Pathology, University of Oxford, Oxford, United Kingdom

## Abstract

**Background:**

Dendritic cells (DC) have been proposed to mediate sexual HIV-1 transmission by capturing the virus in the mucosa and subsequently presenting it to CD4^+ ^T cells. We have demonstrated before that DC subsets expressing higher levels of intercellular adhesion molecule-1 (ICAM-1) are better HIV-1 transmitters. ICAM-1 binds leukocyte function-associated molecule-1 (LFA-1) on T cells, an integrin responsible for adhesion and signaling at the immunological synapse. To corroborate the importance of the ICAM-1— LFA-1 interaction, we performed transmission experiments to LFA-1 negative leukocytes from Leukocyte Adhesion Deficiency type 1 (LAD-1) patients.

**Results:**

We clearly show that DC-mediated HIV-1 transmission to LAD-1 T cells is impaired in comparison to healthy controls. Furthermore, HIV-1 transmission to T cells from a unique LAD-1 patient with a well characterized LFA-1 activation defect was impaired as well, demonstrating that activation of LFA-1 is crucial for efficient transmission. Decreased cell adhesion between DC and LAD-1 T cells could also be illustrated by significantly smaller DC-T cell clusters after HIV-1 transmission.

**Conclusion:**

By making use of LFA-1 defect cells from unique patients, this study provides more insight into the mechanism of HIV-1 transmission by DC. This may offer new treatment options to reduce sexual transmission of HIV-1.

## Background

One of the first cell types encountered by HIV-1 during sexual transmission are intraepithelial and submucosal dendritic cells (DC) [1-3]. DC are professional antigen-presenting cells that sample the environment at sites of pathogen entry. Sentinel immature DC (iDC) develop into mature effector DC (mDC) upon activation by microorganisms or inflammatory signals, and migrate to the draining lymph nodes where they encounter and stimulate naïve Th cells [4,5]. HIV-1 has been proposed to make use of this migratory process, being captured by DC and delivered to the lymph node where the virus is transmitted to CD4^+ ^T cells. In addition to this, DC can facilitate local HIV-1 replication in mucosal T cells [6,7]. HIV-1 transmission by DC takes place via cell-cell contact through an 'infectious synapse' [8,9].

We have shown before that intercellular adhesion molecule-1 (ICAM-1) expression on DC is crucial for HIV-1 transmission to T cells: Monocyte-derived DC subsets that express higher levels of ICAM-1 show higher HIV-1 transmission efficiencies to T cells [8], and transmission by both monocyte-derived DC and DC isolated from blood can be inhibited with blocking antibodies against ICAM-1 [8,10]. During antigen presentation, ICAM-1 expressed by DC binds to T cells via leukocyte function-associated molecule-1 (LFA-1). This interaction plays a key role in the initiation of immune responses by strengthening the adhesion between DC and T cells at the immunological synapse [11-13]. LFA-1 is an integrin composed of the non-covalently bound α L-subunit CD11a and β2-subunit CD18 [14]. Lack of proper β2 expression due to a deletion or mutation in the CD18 gene leads to Leukocyte Adhesion Deficiency type-1 (LAD-1). Patients with this rare recessive disorder suffer from impaired wound healing without pus formation and recurring necrotic soft tissue infections. As CD11/CD18 heterodimers pair intracellularly, LFA-1 is not expressed at the cell surface of leukocytes from LAD-1 patients. The migration of leukocytes from the bloodstream into inflamed tissue is consequently hampered. In healthy individuals, stimulation of rolling leukocytes along endothelial cell lining induces a conformational change of CD11/CD18 heterodimers from a low to a high ligand-binding state, bringing cells to a halt. As expected, this adhesive process is impaired in LAD-1 patients [15-19]. A unique variant of the LAD-1 disorder has been described (LAD-1/variant syndrome) [20]. Cells of this patient with clinical features of a mild LAD-1 disorder do express LFA-1, but cellular activation does not result in activation of LFA-1, i.e. the 'inside-out signaling' that is necessary for increased ICAM-1 binding is impaired [12,20-22].

To further corroborate the importance of LFA-1 in HIV-1 transmission, we made use of T cells from LAD-1 patients. We found that DC-mediated HIV-1 transmission to LFA-1 negative T cells is impaired in comparison to healthy controls. Furthermore, HIV-1 transmission to T cells isolated from the unique LAD-1/variant patient is impaired too, meaning that not only recognition of ICAM-1 but also high-activity binding is important for efficient transmission. Finally, we show that one day after HIV-1 transmission, DC-T cell clusters of LAD-1 and LAD-1/variant cells are significantly smaller than control clusters, which is illustrative for the reduced cell-cell adhesion in LAD-1 patients. By making use of cells isolated from unique patients, this study provides more insight into DC-mediated HIV-1 transmission, which may offer new options to inhibit HIV-1 transmission.

## Results

### DC-mediated transmission to LAD-1 T cells is impaired

**Figure 1:**
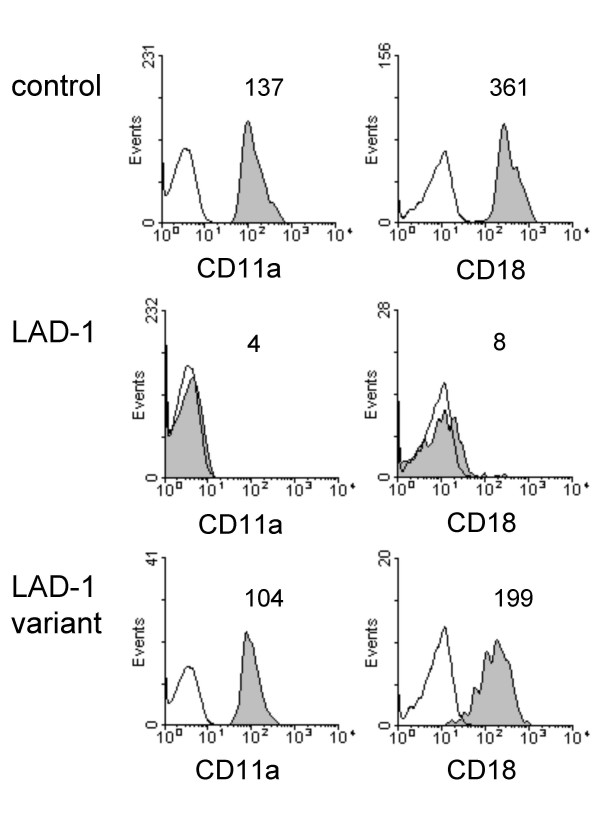
**Phenotype of LAD-1 and control PBL**. Representative FACS staining for CD11a (LFA-1) and CD18 of PBL from one healthy donor (upper panel), a LAD-1 donor (# 1 from Table 1) (middle panel) and a unique patient with a LFA-1 activation defect (LAD-1/variant) (lower panel). The open histograms represent the isotype controls. The mean fluorescence intensity (MFI) is indicated.

To investigate the importance of the ICAM-1— LFA-1 interaction in DC-mediated HIV-1 transmission, we performed transmission experiments with DC obtained from healthy donors and peripheral blood leukocytes (PBL) from LAD-1 patients or healthy controls. We isolated leukocytes from three different LAD-1 patients, whose characteristics are given in Table 1. To confirm the negative LFA-1 status of LAD-1 leukocytes, we performed FACS analysis on CD11a and CD18, of which one representative patient and control are depicted in Fig. 1, upper two panels. We further determined by FACS that the expression of CD4 and CXCR4 was comparable to healthy controls (results not shown). In order to test the transmission efficiency to LAD-1 T cells, we used DC stimulated by poly (I:C) since this subset expresses the highest level of ICAM-1 and is the most efficient HIV-1 transmitter [8].

**Table 1:** Characteristics of LAD patients

	Gender/age	CD11a/CD18 expression (MFI)	% LFA-1 expression	Details
LAD-1 #1	Male, 8 years	4/8	<1% of normal	Late detachment of umbilical cord, recurrent infections, BM transplantation planned.
LAD-1 #2	Male, 15 years	4/8	5% of normal	Recurrent infections, no chemotaxis/adhesion of granulocytes. Received granulocyte transfusions, no BM donor available.
LAD-1 #3	Female, 3 years	4/7	n.d.	Mild symptoms, ready for BM transplantation.
LAD-1 variant	Male, 12 years	104/199	normal	Late detachment of umbilical cord, mild nonpussing inflammatory responses, necrotic of nature (20). Granulocyte transfusions for life-threatening pneumonia. Recently BM transplanted.

Expression of CD11a/CD18 on leukocytes was determined by FACS before addition to DC.

MFI: mean fluorescence intensity. n.d.: not determined. BM: bone marrow

**Figure 2:**
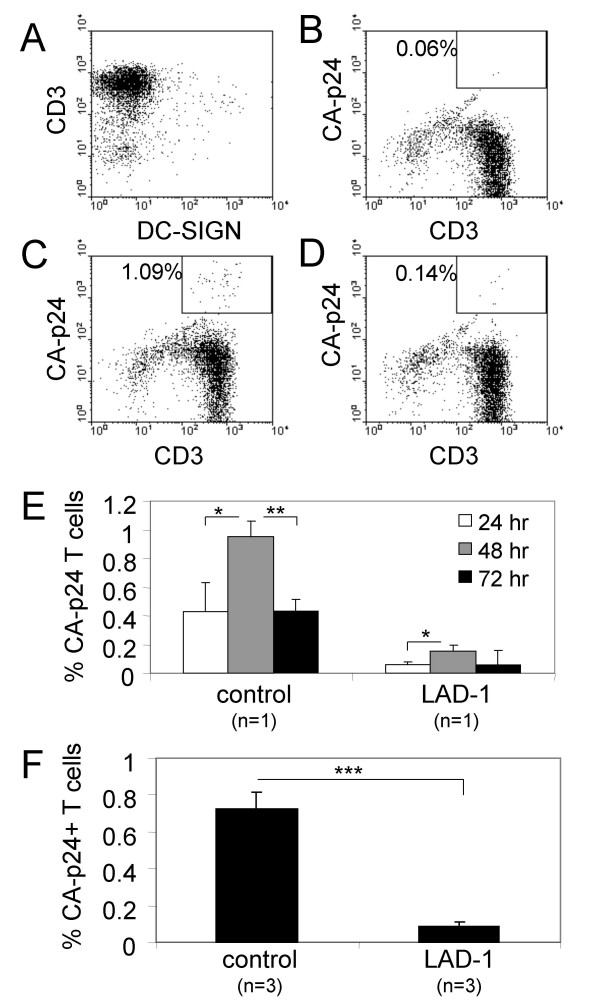
**DC-mediated HIV-1 transmission to LFA-1 negative T cells is impaired**. DC were incubated with HIV-1, followed by washing to remove unbound virus. Subsequently, LAD-1 or control PBL were added to allow transmission of HIV-1. To prevent production of new virions, the cells were cultured in the presence of SQV (single-cycle transmission assay). 2 days after transmission, PBL were harvested and stained for CD3, DC-SIGN and intracellular CA-p24 to determine the transmission efficiency. (A) CD3 and DC-SIGN staining. (B) CA-p24^+ ^CD3^+ ^T cells of an uninfected sample. (C) and (D) Representative FACS staining of a healthy control and a LAD-1 patient, respectively. The percentage CA-p24^+ ^CD3^+ ^cells is indicated. (E) Kinetics of intracellular CA-p24 levels for a representative healthy and LAD-1 donor (n = 1). Error bars represent SD (F) Summary of HIV-1 transmission to T cells of healthy controls (n = 3) and LAD-1 patients (n = 3), two days post transmission. Error bars represent SEM. *P < 0.05, **P < 0.01, ***P < 0.001. One DC donor was used for all transmissions, to reduce variation.

We incubated the DC with HIV-1 for 2 hr, followed by washing steps to remove unbound virus. After addition of LAD-1 or control PBL, we determined the transmission efficiency by measuring the accumulation of HIV-1 capsid protein p24 (CA-p24) in T cells by FACS 1–3 days later. To prevent subsequent rounds of HIV-1 replication after transmission in this single-cycle transmission assay, we added an inhibitor of the viral protease (saquinavir, [23,24]). To distinguish virus transmitted to T cells from HIV in DC, we co-stained with CD3 and DC-SIGN. DC and T cells tend to cluster, which would hamper an accurate estimation of transmission efficiency by FACS. We therefore added EDTA to our FACS buffer to reduce the amount of cell clustering. Indeed, the majority (95%) of the CD3 positive cells was negative for DC-SIGN, showing that only a few DC were attached to T cells during the FACS analysis (Fig. 2A). In uninfected controls of LAD-1 and healthy PBL only background percentages of CA-p24 positive T cells were scored (0.06%, Fig. 2B). Addition of HIV-1 resulted in an increase in CA-p24 positive T cells when using PBL from healthy controls (1.09%, Fig. 2C), whereas only a slight increase was observed in PBL from LAD-1 donors (0.14%, Fig. 2D). The intracellular CA-p24 levels reached a maximum two days after DC-mediated HIV-1 transmission for both control and LAD-1 PBL, which is depicted in Fig. 2E for one representative control and LAD-1 patient. On average, DC-mediated HIV-1 transmission to control T cells was nine times more efficient (Fig. 2F, n = 3).

### HIV-1 replication in LFA-1 negative T cells after DC-mediated transmission is delayed

**Figure 3:**
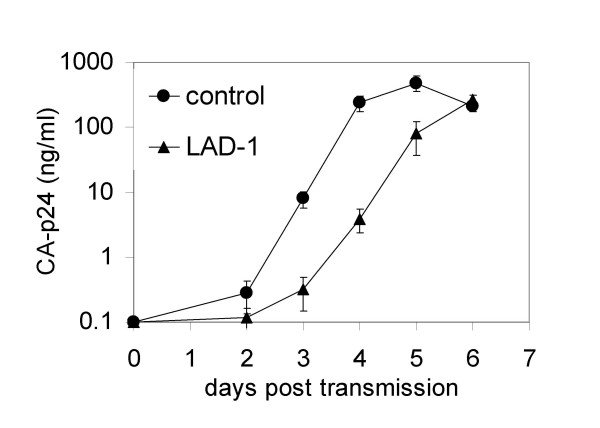
**HIV-1 replication in LFA-1 negative T cells after DC-mediated transmission is delayed**. After DC-HIV incubation and washing, LAD-1 and control PBL were added. Viral replication was followed by measuring CA-p24 production in the supernatant by ELISA. We used cells of three LAD-1 and three healthy donors (n = 3). Error bars represent SEM.

In addition to quantification of the transmission efficiency in a single-cycle transmission assay (Fig. 2), we followed viral replication after transmission (Fig. 3). In this spreading infection assay, we did not add saquinavir to allow cell-cell spread of newly produced virus. The replication of HIV-1 after transmission to LAD-1 PBL is delayed with 1–2 days in comparison to healthy controls (Fig. 2), which reflects the lower transmission efficiency of figure 2. Since CA-p24 levels eventually reach a similar plateau, we conclude that LAD-1 cells are susceptible to HIV-1, but that the transmission is taking place at a lower efficiency.

### Activation of LFA-1 is crucial for efficient HIV-1 transmission

**Figure 4:**
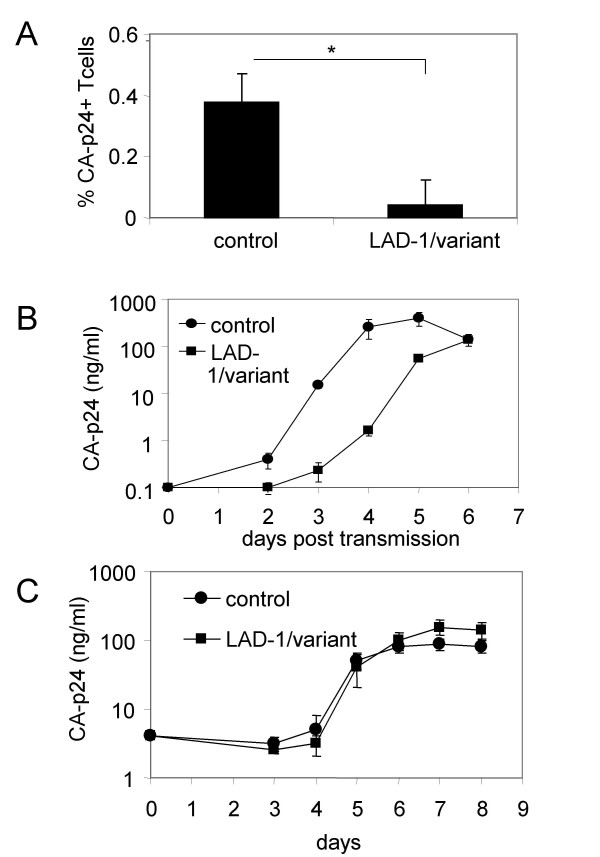
**Activation of LFA-1 is crucial for efficient HIV-1 transmission**. (A) Single-cycle transmission assay. Two days after DC-mediated transmission in the presence of SQV, PBL were harvested and stained for CD3, DC-SIGN and intracellular CA-p24 to determine the percentage of HIV-1 positive T cells. Experiments were performed twice with cells from the same patient isolated on two separate occasions. Cells of two different healthy controls were used. Error bars represent SD. *P < 0.02. (B) Viral replication in T cells after DC-mediated transmission to healthy and LAD-1/variant PBL was followed by CA-p24 ELISA of the supernatant. (C) LAD-1/variant PBL or control cells were stimulated with anti-CD3/CD28 antibodies and were infected with HIV-1. Viral replication was followed by CA-p24 ELISA.

In order to efficiently bind ICAM-1, LFA-1 needs to be activated. Cellular activation by chemokines from endothelial cells or by TCR/CD3 cross-linking results in a conformational change of LFA-1 from a low to a high ligand-binding state [12,21,25]. We questioned whether LFA-1 expression by T cells is sufficient for efficient HIV-1 transmission by DC, or that additional activation of LFA-1 is necessary. To investigate this, we used cells from a unique patient with mild LAD-1 symptoms (LAD-1/variant). The leukocytes from this patient express LFA-1 (Fig. 1 and Table 1), but the integrin cannot be induced into an active conformation [20]. In comparison to the experiments with LFA-1 negative cells, we obtained similar results: HIV-1 transmission to LAD-1/variant T cells was impaired in the single-cycle assay (Fig. 4A) and replication after transmission was delayed with 2 days (Fig. 4B). This demonstrates that LFA-1 has to be activated to a high ligand-binding state in order to mediate HIV-1 transmission. As an additional control, we infected LAD-1/variant PBL with HIV-1 in the absence of DC (Fig 4C). HIV-1 replication in CD3/CD28 stimulated PBL from the LAD-1/variant patient was comparable to healthy controls, showing that not replication but efficient transmission by DC depends on LFA-1 activation.

### LAD-1 and LAD-1/variant T cells form smaller clusters with DC

**Figure 5:**
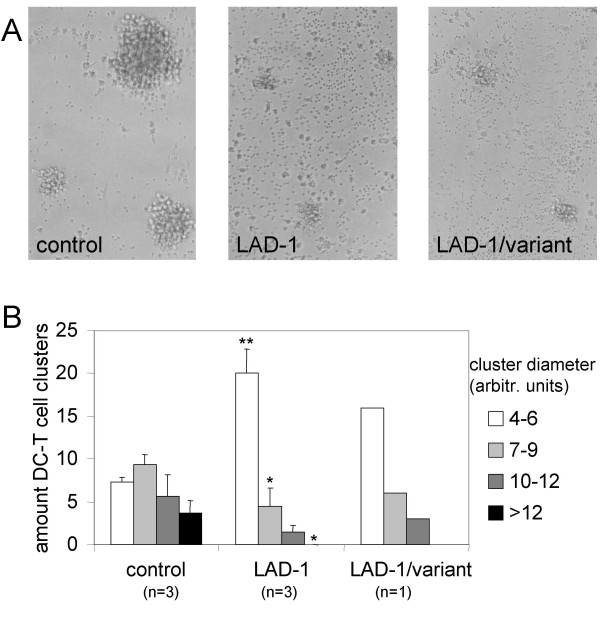
**LAD-1 and LAD-1/variant T cells form smaller clusters with DC**. (A) Representative light microscopic images of DC-T cell clusters with healthy, LAD-1 and LAD-1/variant T cells, one day after HIV-1 transmission. (B) The number and diameter of DC-T cell clusters of cells from LAD-1 patients (n = 3), the LAD-1/variant patient (n = 1), and healthy controls (n = 3) were determined one day after HIV-1 transmission, and the clusters were subsequently grouped according to size. Error bars represent standard deviations. *P < 0.05, **P < 0.01, compared to corresponding cluster group from 'control'.

DC attract T cells and form large clusters *in vivo *and *in vitro*, a process that is dependent on cell-cell adhesion [26,27]. We studied the clusters of DC and leukocytes 24 hr after DC-mediated HIV-1 transmission. The cluster size of DC with T cells from healthy individuals was clearly larger than the clusters with T cells from LAD-1 and LAD-1/variant T cells, as is shown in the photographs (Fig 5A). Quantitative determination of the amount and diameter of clusters showed that although the number of clusters was only slightly reduced (30, 26 and 25 clusters on average for control, LAD-1 and LAD-1/variant respectively), the mean cluster diameter of control cells was significantly larger (9.1 versus 5.6 and 6.2 in arbitrary units for LAD-1 and the variant respectively; p < 0.001 and <0.002). We subsequently grouped the clusters according to diameter (Fig. 5B), and clearly demonstrate that their actual number is not reduced for the LAD-1 and LAD-1/variant T cells, but that they are significantly smaller in size.

## Discussion

In the present study, we demonstrate the role of LFA-1 in HIV-1 transmission by DC. Previously we have shown that ICAM-1 expression on both monocyte-derived DC and DC from blood is critical for HIV-1 transmission [8,10]. In accordance with this, we now show that DC-mediated transmission to LFA-1-negative T cells from LAD-1 patients is severely impaired. Normally, LFA-1 is activated by different kinds of stimuli, and binding to ICAM-1 is subsequently up-regulated (inside-out signaling) [12,13,21,28]. Currently, it is assumed that activation of LFA-1 may be regulated via changes in affinity (active conformation), avidity (clustering) or both [22,29-33]. The fact that transmission to T cells of a unique patient (LAD-1/variant syndrome) [20], with an inside-out signaling deficient LFA-1, was impaired as well, demonstrates for the first time that LFA-1 activation is crucial for DC-mediated HIV-1 transmission. Although LFA-1 of this patient is able to recognize its ligand, no high avidity/affinity binding to ICAM-1 is taking place. Since there is no strong binding to ICAM-1, signaling through LFA-1 into the T cell (outside-in signaling) is probably not taking place either. In healthy individuals, signaling through LFA-1 after ICAM-1 binding leads to actin polymerization and remodeling, which is important for enhanced cell adhesion [34]. Impaired cell adhesion in LAD-1 (and variant) patients can also be illustrated by the significantly smaller clusters of DC with T cells (Fig. 5). A smaller number of T cells that is tightly attached to DC will result in a decrease of the window of opportunity for HIV-1 transmission. Furthermore, it is likely that the creation of an 'infectious synapse' is disturbed in LAD-1 and LAD-1/variant patients. Others have shown that DC-SIGN is an important component of the infectious synapse [9,35]. Our results strongly indicate that LFA-1 is also important for infectious synapse formation, possibly through cytoskeletal rearrangements that are induced by ICAM-1 binding.

The infectivity and subsequent replication of HIV-1 in T cells can be influenced by T cell activation and proliferation. Due to the young age of the patients and severity of the disease, no more cells could be obtained from these patients to perform a separate mixed lymphocyte reaction (MLR). However, we found no lower cellular proliferation of LAD-1 and LAD-1/variant T cells after co-culture with DC during FACS analysis, nor did we find higher percentages of dead cells. In addition, the leukocytes of the LAD-1/variant patient have been shown to proliferate normally, and have normal calcium influx, actin metabolism and protein kinase activity [20]. Another factor influencing HIV-1 infectivity is the incorporation of host ICAM-1 in budding virions and expression of LFA-1 on target cells [36-40]. To critically test this hypothesis, we performed transmission experiments with HIV-1 produced both in C33A cells and in PM1 T cells. C33A cells do not express ICAM-1 (or LFA-1), yielding virions without ICAM-1. With both virus stocks, we found impaired DC-mediated transmission to T cells of LAD-1/variant and LAD-1 patients, ruling out that the virus-producer cell is of influence. This observation is in concordance with our previous work [8] and the work of Bounou and co-workers, who showed that in DC-mediated HIV-1 transmission, virion-associated ICAM-1 is of no influence [41]. Furthermore, we have shown that LAD-1/variant and control T cells are equally susceptible to HIV-1 in the absence of DC, demonstrating that the DC-mediated transmission itself is impaired, instead of the ability of HIV-1 to infect these cells.

The importance of the ICAM-1— LFA-1 interaction for DC-T cell contact and HIV-1 transmission suggests a new therapeutic target for the development of transmission-blockers. Interestingly, the fungal metabolite lovastatin, which belongs to the statin compounds used in the treatment of hypercholesterolemia, was shown to bind LFA-1 and inhibit the interaction with ICAM-1 [42]. Furthermore, lovastatin was recently shown to block entry of ICAM-1-containing HIV-1 virion particles into T cells [43]. We therefore tested whether lovastatin could block DC-mediated HIV-1 transmission. Although we measured a significant decrease in HIV-1 transmission, inhibition was due to toxicity of the compound at the micromolar range that is required for blocking the ICAM-1— LFA-1 interaction (results not shown). Given the importance of LFA-1 in HIV-1 transmission by DC, future research should focus on the development of less toxic derivatives or other small molecule inhibitors of the ICAM-1— LFA-1 interaction [44,45]. Now that there is proof that compounds can be generated that potently inhibit and target integrins like LFA-1 [46] the use of such selective oral compounds may prove very useful in preventing or treating various diseases. With respect to HIV-1 transmission, these compounds can be used in combination with other drugs in a microbicide mixture that will help slowing down the ongoing HIV-1 pandemic.

## Materials and methods

### Generation of monocyte-derived dendritic cells

Peripheral blood mononuclear cells (PBMC) were isolated from blood of healthy donors by density centrifugation on Lymphoprep (Nycomed, Torshov, Norway). Subsequently, PBMC were layered on a Percoll gradient (Pharmacia, Uppsala, Sweden) with three density layers (1.076, 1.059, and 1.045 g/ml). The light fraction with predominantly monocytes was collected, washed, and seeded in 24-well or 6-well culture plates (Costar, Cambridge, MA, USA) at a density of 5 × 10^5 ^cells or 2,5 × 10^6 ^per well, respectively. After 60 min at 37°C, nonadherent cells were removed, and adherent cells were cultured to obtain immature DC in Iscove's modified Dulbecco's medium (IMDM; Life Technologies Ltd., Paisley, United Kingdom) with gentamicin (86 μg/ml; Duchefa, Haarlem, The Netherlands) and 10% fetal clone serum (HyClone, Logan, UT, USA) and supplemented with GM-CSF (500 U/ml; Schering-Plough, Uden, The Netherlands) and IL-4 (250 U/ml; Strathmann Biotec AG, Hannover, Germany). At day 3, the culture medium with supplements was refreshed. At day 6, maturation was induced by culturing the cells with poly (I:C) (20 μg/ml; Sigma-Aldrich, St. Louis, MO, USA). After two days, mature CD14^- ^CD1b^+ ^CD83^+ ^DC were obtained. All subsequent tests were performed after harvesting and extensive washing of the cells to remove all factors. Mature DC were analysed for the expression of cell surface molecules by FACS. Mouse anti-human mAbs were used against the following molecules: CD14 (BD Biosciences, San Jose, CA, USA), CD1b (Diaclone, Besançon, France), CD83 (Immunotech, Marseille, France) and ICAM-1 (CD54) (Pelicluster, Sanquin, Amsterdam, The Netherlands). All mAb incubations were followed by incubation with FITC-conjugated goat F(ab')_2 _anti-mouse IgG and IgM (Jackson ImmunoResearch Laboratories, West Grove, PA, USA). Samples were analysed on a FACScan (BD Biosciences).

### Peripheral Blood Leukocytes

Peripheral Blood Leukocytes (PBL) were isolated by layering PBMC from healthy donors and LAD-1 patients on a Percoll gradient. The heavy fraction with predominantly PBL was collected and stored at -150°C. PBL were cultured in IMDM with 10% FCS, gentamycin, 10 U/ml IL-2 (Cetus, Emeryville, CA, USA) and *Staphylococcus enterotoxin B *(SEB; Sigma-Aldrich; final concentration, 10 pg/ml). Mouse mAb to human CD28 (CLB-CD28/1) and human CD3 (CLB-T3/4E-1XE) were obtained from Sanquin (Amsterdam, The Netherlands).

### Virus stocks

C33A cervix carcinoma cells or PM1 T cells were transfected using calcium phosphate or electroporation respectively with 5 μg of the molecular clone of HIV-1 LAI. Since any of the patients could bear one or two mutant alleles for the CCR5 co-receptor, resulting is decreased susceptibility to CCR5-using HIV-1, we chose to use CXCR4-using HIV-1 LAI. The virus containing supernatant was harvested 3 to 5 days post transfection, filtered and stored at -80°C. The concentration of virus was determined by CA-p24 ELISA. C33A and PM1 cells were maintained in Dulbecco's Modified Eagle's Medium (DMEM) or Roswell Park Memorial Institute (RPMI) medium 1640 (Life Technologies) respectively, both supplemented with 10% FCS, 2 mM sodium pyruvate, 10 mM HEPES, 2 mM L-glutamine, penicillin (100 U/ml) (Sigma-Aldrich) and streptomycin (100 μg/ml) (Invitrogen, Breda, The Netherlands).

### HIV transmission assay and CA-p24 measurement

Fully matured DC were incubated in a 96-well-plate (40–50 × 10^3 ^DC/50 μl/well) with PM1 produced virus (10 ng CA-p24/well) or C33A produced virus (20 ng) for 2 hr at 37°C. The DC were washed with PBS after centrifugation at 400 × g to remove unbound virus. Washing was repeated, followed by addition of 50 × 10^3 ^PBL. Prior to addition to DC the PBL were analyzed by FACS with the following mouse anti-human antibodies: FITC-labeled CD11a (Pelicluster, Sanquin), APC-labeled CD4 (BD Biosciences) and PE-labeled CXCR4 (BD Biosciences). CD18 (Pelicluster, Sanquin) incubation was followed by incubation with FITC-conjugated goat F(ab')_2 _anti-mouse IgG and IgM (Jackson ImmunoResearch Laboratories). Viral replication after transmission was followed by measuring CA-p24 in the culture supernatant by ELISA. To determine intracellular CA-p24 in the single-cycle transmission assay, saquinavir (Roche, London, United Kingdom at 0.2 μM) was added to prevent cell-to-cell spread of newly produced virions. After 48 hr, the cells were harvested and stained with FITC-labeled CD3 (BD Biosciences) and APC-labeled DC-SIGN (R7D Systems, MN, USA), followed by fixation with 4% PFA and washing with washing buffer (PBS with 2 mM EDTA and 0.5% BSA). Fixated cells were then washed with perm/wash buffer (BD Biosciences), and incubated with PE-labelled CA-p24 (KC57-RD1, Coulter, Hialeah, FL, USA) followed by washing with successively perm/wash- and washing buffer. Cells were then analysed by FACS.

### Statistical analysis

Data were analysed for statistical significance (GraphPad InStat, Inc, San Diego, CA, USA) using ANOVA. A *p *value <0.05 was considered to be significant.

## Abbreviations

ICAM-1: intercellular adhesion molecule-1; LAD-1: Leukocyte Adhesion Deficiency type 1; LFA-1: leukocyte function-associated molecule-1
